# Utility, validity, feasibility and acceptability of a clinician-administered depression, two-question screening tool for routine multiple sclerosis clinic administration

**DOI:** 10.1177/13524585261435415

**Published:** 2026-04-26

**Authors:** Lisa Grech, Michelle Allan, David Skvarc, Ruby Hamer, Emily Friedel, Victor Chong, Andrew Giles, Jayashri Kulkarni, Jennifer Neil, Deepa Rajendran, Nevin John, Martin Short, Sally Shaw, Nigel Caswell, Vanessa Fanning, Ernest Butler

**Affiliations:** School of Psychology, Deakin University, Burwood, VIC, Australia; Department of Medicine, Monash Health, Monash University, Clayton, VIC, Australia; Department of Medicine, Monash Health, Monash University, Clayton, VIC, Australia; Frankston Neurology, Frankston, VIC, Australia; School of Psychology, Deakin University, Burwood, VIC, Australia; School of Psychology, Deakin University, Burwood, VIC, Australia; Department of Psychological Sciences, Swinburne University of Technology, Hawthorn, VIC, Australia; School of Psychology, Deakin University, Burwood, VIC, Australia; Department of Medicine, Monash Health, Monash University, Clayton, VIC, Australia; Neurological Alliance Australia, Multiple Sclerosis Australia, North Sydney, NSW, Australia; Department of Psychiatry, Monash Alfred Psychiatry Research Centre, Monash University, Melbourne, VIC, Australia; Monash School of Medicine, Monash University, Melbourne, VIC, Australia; Department of Medicine, Monash Health, Monash University, Clayton, VIC, Australia; Department of Neurology, Monash Medical Centre, Monash Health, Clayton, VIC, Australia; Department of Medicine, Monash Health, Monash University, Clayton, VIC, Australia; Department of Neurology, Monash Medical Centre, Monash Health, Clayton, VIC, Australia; Department of Neurology, Monash Medical Centre, Monash Health, Clayton, VIC, Australia; Psychology, Livology, Blackburn, VIC, Australia; People with MS Victoria, Melbourne, VIC, Australia; People with MS Victoria, Melbourne, VIC, Australia; Department of Medicine, Monash Health, Monash University, Clayton, VIC, Australia; Frankston Neurology, Frankston, VIC, Australia; Department of Neurology, Peninsula Health, Frankston, VIC, Australia

**Keywords:** Validity, specificity, sensitivity, multiple sclerosis, depression, Two-Question Screening tool, 2QS

## Abstract

**Background:**

Depression is prevalent among individuals with multiple sclerosis (MS) yet frequently goes undetected and untreated. Time constraints are a barrier to depression screening in MS clinics. We evaluated the clinical utility, diagnostic accuracy and feasibility of the Two-Question Screening tool (2QS) for routine, in-clinic depression screening.

**Objectives:**

A prospective cross-sectional study of 207 consecutively recruited adults with MS (M*age* = 47.3 ± 12.7, 77.3% female) was conducted at a metropolitan MS Clinic. Clinicians administered the 2QS during in-clinic or telehealth consultations. To assess the sensitivity and specificity of the 2QS in identifying depression, participants underwent a Structured Clinical Interview for *Diagnostic and Statistical Manual for Mental Disorders – Fifth Edition* (SCID-5) for major depressive disorder (MDD), Patient Health Questionnaire-9 (PHQ-9), and the Depression, Anxiety and Stress Scale-21 (DASS-21). Internal consistency, convergent validity and clinician feasibility were assessed.

**Results:**

The 2QS had 100% (95% CI: 71%–100%) sensitivity and 68% (95% CI: 60%–76%) specificity for detecting MDD. Clinician screening adherence was 76%. For the in-clinic subsample, clinician-administered 2QS correlations were SCID-5 MDD, *r* = 0.39 (*r* = 0.60 with subthreshold depression symptoms added); PHQ-9, *r* = 0.73; and DASS-D, *r* = 0.74.

**Conclusions:**

Clinician-administered 2QS is valid and feasible for routine depression screening at MS clinic appointments. With high sensitivity and acceptable specificity, the clinician-administered 2QS is suitable to improve depression detection in people with MS.

## Introduction

Multiple sclerosis (MS) is the most common neurodegenerative disease affecting young adults.^1^ It has broad-ranging symptoms, including muscle weakness, reduced coordination, spasticity, fatigue, dysphagia, bladder and bowel dysfunction, cognitive impairment and emotional difficulties.^1^ Depression is common in people with MS, with estimates of lifetime prevalence ranging up to 50%^2^ and an annual prevalence of ~26%.^3,4^

In MS, depression results from biological and psychosocial factors. Biological factors include frontotemporal and hippocampal structural abnormalities and a functional disconnect between limbic and frontal regions.^5^ Furthermore, structural magnetic resonance imaging has revealed associations between MS pathology and depression symptoms,^6^ while other findings support the relationship between neuroinflammation in MS and depression.^7^ Psychosocially, factors associated with depression in MS include disease uncertainty, reduced social support, lower mobility, poorer vocational and educational outcomes and increased substance abuse.^5,8^

Untreated depression has substantial consequences for people with MS, including higher perceived symptom severity, reduced disease self-management and medication adherence, reduced psychological wellbeing and quality of life, increased social isolation and suicide ideation.^5,9–11^ Yet, studies show that clinically significant depressive symptoms go undetected in 11.9%–35.8% and under-treated in 21.0%–65.0% of people with MS.^12–17^ Given the consequences of MS depression, guidelines have recommended routine depression screening.^18,19^

Improving MS depression detection requires identifying tools suitable for routine screening in busy healthcare settings. Our qualitative research identified time as a key clinician barrier to improved mental health care for people with MS.^20^ Therefore, depression screens that are practical for routine neurological practice must be highly efficient in administration speed and diagnostically accurate. The 2-Question Screen (2QS), derived from the Primary Care Evaluation of Mental Disorders (PRIME-MD),^21^ may meet both criteria. In 260 people with MS, the 2QS displayed 98.5% sensitivity and 87% specificity, with half of the 13% who were assessed as false positives having subthreshold symptoms likely to benefit from monitoring/follow-up.^22^ However, research on the suitability of the 2QS in clinical settings is lacking.

The current study aimed to assess the criterion validity, feasibility and acceptability of the clinician-administered 2QS to assess depression in MS. We aimed to determine the following 2QS psychometric properties: (1) sensitivity, specificity and feasibility when delivered by clinicians as part of routine clinical consultations and (2) validity, diagnostic utility and acceptability for depression screening in people with MS.

## Methods

The study design adhered to the Statement for Reporting Studies of Diagnostic Accuracy (STARD) guidelines.^23^

### Research design and participants

This cross-sectional research recruited participants with MS (*N* = 207) through a large metropolitan hospital MS clinic between 12 June 2022 and 22 June 2023. People with a confirmed MS diagnosis, aged 18 or older, with a scheduled MS clinic appointment were invited to participate and recruited consecutively. Exclusion criteria included the presence of a comorbid neurological condition, inability to read and converse in English or being deemed too medically unwell to contact.

### Measures

A 75-item survey was developed, which included demographic (age, gender, education, employment status), disease-related (MS duration, subtype, mobility, MS appointment frequency) and mental health questions (history of mental health conditions, current perceived depression, medically diagnosed depression, depression treatment). This research was part of a broader study assessing mental health symptoms in people with MS. Only depression symptoms measured with the below screening tools are reported here. (See Supplementary Information for a complete survey.)

The 2QS^21^ has two questions: (1) ‘During the past month, have you often been bothered by feeling down, depressed or hopeless?’ and (2) ‘During the past month, have you often been bothered by little interest or pleasure in doing things?’ Responding ‘yes’ to either question is considered a positive screen.^21^ These two questions are consistent with the *Diagnostic and Statistical Manual for Mental Disorders – Fifth Edition* (*DSM*-5), which requires the presence of low mood or anhedonia in major depressive disorder (MDD).^24^

The Patient Health Questionnaire (PHQ-9)^25^ was administered to measure depression severity. The nine items of the PHQ-9 parallel the nine symptom categories listed in the *DSM*-5 for MDD criteria:^24^ loss of interest, feeling depressed, sleep problems, loss of energy, appetite problems, self-blame, concentration problems, agitation/retardation, suicidal ideation. Each symptom is rated on a 4-point Likert-type scale ranging between 0 (not at all) and 3 (nearly every day). Scores range between 0 and 27, with 0–4 being no symptoms, 5–9 mild, 10–14 moderate, 15–19 moderately severe and 20+ severe. The PHQ-9 has been extensively used in MS research and has 95.0% sensitivity and 88.3% specificity to identify or exclude MDD in people with MS using a cutoff score of 11.^26^

Depression, Anxiety and Stress Scale – Twenty-One (DASS-21) is freely available and broadly used in clinical practice. It has three subscales that measure depression, anxiety and stress symptoms, with seven items each rated on a 4-point Likert-type scale ranging from 0 (never) to 3 (almost always) over the past 7 days. Scores for each subscale range from 0 to 21 and are categorised as nil, mild, moderate, severe and extremely severe. The DASS-21 has good concurrent and predictive validity assessing symptoms of depression, anxiety and stress in people with MS.^27^

Structured Clinical Interview for DSM-5 (SCID-5) – Depression Module was used as the gold standard diagnostic interview to assess for the current presence of MDD and subthreshold depression symptoms. The SCID-5 is a semi-structured interview guide for diagnosing MDD, consistent with *DSM*-5 diagnostic criteria, and has shown high agreement with clinical diagnoses, excellent reliability and high specificity.^28^

Patient Determined Disease Steps (PDDS) is a self-reported measure of mobility/disability in people with MS. The PDDS has nine levels, ranging from 0 (no disability) to 8 (bedridden). It has demonstrated good convergent validity with the Expanded Disability Status Scale (ρ = 0.78) and the Multiple Sclerosis Walking Scale-12 (ρ = 0.80).^29^

### Procedure

Consenting participants were requested to attend an hour prior to or following their appointment to complete the assessment questionnaire on an electronic tablet device in the waiting room (~15 minutes) and undergo assessment by a research team member using the SCID-5 MDD module (~30 minutes). Participants attending via telehealth were emailed a link to the assessment questionnaire to complete remotely, and a video platform assessment was conducted on the day of their clinic appointment. Neurologists and MS nurses administered the 2QS to consenting participants during consultations and entered the results for each participant into an electronic capture form. Administering clinicians did not have access to SCID-5 MDD results. The participant’s nominated health professional was contacted in writing by the research team if the participant reported moderate or severe depression symptoms.

### Analysis

An a priori power analysis determined that a sample of 208 was required for assessment of diagnostic accuracy using alpha = 0.05, a predetermined estimate of sensitivity and specificity of 0.90 and an error margin of 0.08.^30^ Following data cleaning, questionnaire scales and subscales were summed. Missing data were assessed for randomness; no specific patterns were identified, and replacement values were not imputed. Where required, data categories were collapsed for analysis. This included combining regional and rural location, combining primary and secondary MS subtypes into a ‘progressive MS’ category and combining moderate and severe self-perceived depression. To characterise the sample, chi-square tests and *t*-tests were used to identify significant between-group demographic differences, and the McNemar test was conducted to assess differences between clinician- and electronically-administered 2QS responses.

Sensitivity, specificity, positive and negative predictive values, positive and negative likelihood ratios and accuracy were calculated using MedCalc’s diagnostic evaluation calculator: https://www.medcalc.org/calc/diagnostic_test.php for binary variables with a point prevalence of depression set at 26%, consistent with previous research.^3,4^ Receiver Operating Characteristic analysis was undertaken to determine area under the curve for continuous variables.

Implementation feasibility for clinicians administering the 2QS in consultations was determined by the frequency of clinician-administered 2QS for consenting participants, with a total of 70% screened considered feasible. For hypothesis testing, alpha was set at <0.05. Unless otherwise stated, statistical analysis was undertaken with the Statistical Package for Social Sciences (SPSS) software, version 30.

The study was registered with ANZCTR: ACTRN12622000543785. Ethics approval for this study was obtained from Monash Health Human Research Ethics Committee (REF:37682, LOCALREF: RES-21-0000-740A).

## Results

### Demographics

Demographic and disease characteristics are presented in Table 1. For demographic and disease-related factors, only gender differed significantly between in-clinic and telehealth participants, with a higher proportion of females seen in the clinic. The sample comprised 207 participants (see Figure S1 for STARD flow diagram) with MS (mean age = 47.3 ± 12.7 years, 77.3% female). Participants most commonly reported relapsing remitting MS subtype (73.8%) and nil-to-mild disability level (52.1%).

**Table 1.** Demographic and disease characteristics of participants with MS.

Characteristic	Total *n* = 207 (%)	In clinic *n* = 133 (64.3)	Telehealth *n* = 74 (35.7)	χ^2^/*t*-test *p*-value
**Demographics**				
**Gender**				
Male	47 (22.7)	36 (14.9)	11 (27.1)	
Female	160 (77.3)	97 (85.1)	63 (72.9)	0.045
**Age**: mean (SD)	47.3 (12.7)	47.4 (12.8)	47.1 (12.7)	0.883
**Highest education**				
Primary/Secondary school	61 (31.3)	40 (31.0)	21 (31.8)	
Vocational/Trade/Diploma	54 (27.7)	41 (31.8)	13 (19.7)	
Undergraduate university	44 (22.6)	29 (22.5)	15 (22.7)	
Postgraduate university	36 (18.5)	19 (14.7)	17 (25.8)	0.158
**Occupation**				
Full-time	67 (35.3)	21 (32.8)	46 (36.5)	
Part-time	54 (28.4)	19 (29.7)	35 (27.8)	
Retired (including medically retired)	45 (23.7)	15 (14.9)	30 (23.8)	
Other	24 (12.6)	9 (19.1)	15 (11.9)	0.945
**Metropolitan/Regional/Rural**				
Metropolitan	156 (81.3)	109 (84.5)	47 (74.6)	
Regional	3 (1.6)	2 (1.6)	1 (1.6)	
Rural	33 (17.2)	18 (14.0)	15 (23.8)	0.099^a^
**MS related**				
**MS subtype**				
Relapsing-remitting MS (RRMS)	144 (73.8)	92 (71.3)	52 (78.8)	
Secondary progressive MS (SPMS)	13 (6.7)	7 (5.4)	6 (9.1)	
Primary progressive MS (PPMS)	9 (4.6)	8 (6.2)	1 (1.5)	
Other/Don’t know	29 (14.9)	22 (17.1)	7 (10.6)	0.415^b^
**MS duration Years**: mean (SD)	12.7 (10.1)	12.9 (9.7)	12.6 (10.3)	0.870
**PDDS**				
Nil-mild disability	100 (52.1)	72 (56.3)	28 (43.8)	
Moderate disability	48 (25.0)	33 (25.8)	15 (23.4)	
Severe disability	44 (22.4)	23 (18.0)	21 (32.8)	0.065
**Clinic visit frequency**				
3 months	10 (5.1)	5 (3.9)	5 (7.6)	
6 months	129 (66.2)	86 (66.7)	43 (65.2)	
>6 to 12 months	49 (25.1)	31 (24.0)	18 (27.3)	
>12 months	7 (3.6)	7 (5.4)	0 (0.0)	0.176^c^

a χ^2^ Test was performed on two location groups: metropolitan and regional/rural combined, as regional violated the required expected count ⩾5.

b χ^2^ Test was performed on three groups only: relapsing remitting MS, progressive MS (PPMS and SPMS combined) and don’t know, as PPMS violated the required expected count ⩾5.

c View with caution, due to χ^2^ violation of required expected count ⩾5 in three cells.

### Depression

As Table 2 shows, 37.4% of participants self-evaluated their current depressive symptoms as mild or greater, and 28.2% reported receiving treatment for depression, with antidepressant medication being the most common. When assessed using the SCID-MDD interview, 7.8% of the sample met MDD criteria, and an additional 8.8% met subthreshold criteria.

**Table 2.** Current and mental health history of participants with MS.

Mental health	Total *n* = 207 (%)	In clinic *n* = 133 (64.3)	Telehealth *n* = 74 (35.7)	χ^2^/*t*-test *p*-value
**Self-reported current depression, *n* =** **195**				
Yes, mild	42 (21.5)	34 (26.4)	8 (12.1)	
Yes, moderate	26 (13.3)	19 (14.7)	7 (10.6)	
Yes, severe	5 (2.6)	2 (1.6)	3 (4.5)	
No	122 (62.6)	74 (57.8)	48 (72.7)	0.055^a^
**Current mental health treatment** *n* **=** **195**				
Yes, antidepressant	37 (19.0)	25 (19.4)	12 (18.2)	
Yes, therapy	10 (5.1)	6 (4.7)	4 (6.1)	
Yes, antidepressant and therapy	8 (4.1)	6 (4.7)	2 (3.0)	
No	140 (71.8)	92 (71.3)	48 (72.7)	0.836^b^
**2QS clinician administered** *n* **=** **157**				
2QS-1 Yes	54 (34.4)	39 (36.4)	15 (30.0)	0.428
2QS-2 Yes	46 (29.3)	34 (31.8)	12 (24.0)	0.319
2QS Yes Both	38 (24.2)	28 (26.2)	10 (20.0)	0.401
2QS Yes Any (= positive screen)	61 (38.9)	44 (41.1)	17 (34.0)	0.394
**2QS Tablet or Online Survey** *n* **=** **195**				
2QS-1 Yes	88 (45.1)	65 (50.4)	23 (34.8)	0.039
2QS-2 Yes	83 (42.6)	66 (51.2)	17 (25.8)	<0.001
2QS Yes Both	71 (36.4)	54 (41.9)	17 (25.8)	0.027
2QS Yes Any (= positive screen)	100 (51.3)	77 (59.7)	23 (34.8)	0.001
**SCID Depression** *n* **=** **193**				
SCID-MDD	15 (7.8)	12 (9.7)	3 (4.3)	0.185
SCID-MDD + Subthreshold	32 (16.6)	26 (21.0)	6 (8.7)	0.028

a Moderate and severe were combined for χ^2^ analysis to avoid violation of expected cell count requirements.

b χ^2^ Test was performed on two treatment groups only: yes and no, to avoid violation of expected cell count requirements.

Of participants who self-evaluated as currently experiencing nil or mild depression symptoms, 35.9% answered yes to at least one 2QS question when administered via survey, and 24.1% answered yes to at least one 2QS question when a clinician administered.

#### Two-Question Screening tool

##### Clinician-administered versus electronic assessment

When responding to the clinician-administered 2QS, 34.4% of participants responded yes to item 1, and 29.3% responded yes to item 2, totalling 38.9% who responded yes to either question, and 24.2% who responded yes to both. When responding to the electronically-administered 2QS, 45.1% of participants responded yes to item 1, 42.6% to item 2, equating to 51.3% responding yes to either item and 36.4% responding yes to both.

Participants were significantly more likely to respond ‘yes’ to either of the 2QS questions administered via electronic survey than when asked by the clinician. For 2QS-1, 18.1% (*n* = 17) responded ‘yes’ electronically but ‘no’ to the clinician, compared to 7.2% (*n* = 6) who responded ‘yes’ to the clinician, but ‘no’ electronically, χ^2^ = (1, *n* = 145) 4.35, *p* < 0.035, *ϕ* = 0.17. Participants responded ‘yes’ to 2QS-2 electronically, but ‘no’ when asked by the clinician 18.4% (*n* = 19) of the time compared with 3.4% (*n* = 3) who responded ‘yes’ to the clinician and ‘no’ electronically, χ^2^ = (1,145) 10.53, *p* < 0.001, *ϕ* = 0.27.

When a positive screen was considered (‘yes’ to either question), participants were likely to receive a positive screen based on electronic but not clinician responses 13.8% (*n* = 20) of the time, compared with a positive response based on clinician responses but not electronic responses 4.1% (*n* = 6) of the time χ^2^ = (1, *n* = 145) 6.50, *p* < 0.011, *ϕ* = 0.21. When participants responded ‘yes’ to both 2QS questions, an electronically recorded ‘yes’ response but clinician administered ‘no’ response occurred 11.0% (*n* = 16) of the time, compared with a ‘yes’ response when clinician administered but ‘no’ electronically 1.4% (*n* = 2) of the time, χ^2^ = (1,145) 9.39, *p* = 0.001, *ϕ* = 0.25.

When clinician administered, the overall accuracy for the 2QS to detect MDD was 77%. This increased slightly to 80% if subthreshold symptoms were included. Sensitivity was 100% using MDD diagnosis and slightly lower when subthreshold symptoms were added (93%). While specificity was reduced, the addition of subthreshold depression symptoms improved specificity from 68% for MDD to 76%.

When the 2QS was electronically completed in the current study, sensitivity was lower for MDD than for MDD and subthreshold symptoms (93% and 97%, respectively), and specificity was further reduced for both (55% and 61%, respectively).

##### In-clinic versus telehealth assessment

Participants completing the electronic survey in clinic were significantly more likely to respond ‘yes’ to either item than participants responding to the survey online and were also significantly more likely to respond ‘yes’ to both items (Table 2). Given these differences, a separate analysis was undertaken for in-clinic and telehealth appointment groups for a positive 2QS. For in-clinic appointments, a significantly higher proportion of participants had a positive 2QS screen when responding via survey administration compared to clinician administration, χ^2^ = (1,103) 11.53, *p* = 0.001, *ϕ* = 0.33, whereas there was no significant difference between survey and clinician administration for telehealth, χ^2^ = (1,42) 0.00, *p* = 1.00.

##### Diagnostic utility

Accuracy, sensitivity and specificity of the 2QS were assessed against the gold standard SCID-5- MDD interview (Table S1). Clinician-administered diagnostic accuracy was higher than electronic survey administration overall. 2QS-1 provided adequate diagnostic accuracy of 80% (95% CI: 72%–86%) for detecting MDD (100% sensitivity [95% CI: 75%–100%], 73% specificity [95% CI: 64%–80%]) while a positive response to any 2QS question provided 77% (95% CI: 69%–83%) accuracy, with excellent sensitivity (100% [95% CI: 71%–100%]) and reduced specificity (68% [95% CI: 60%–76%]). When including subthreshold symptoms of MDD, accuracy, sensitivity and specificity were acceptable for the clinician-administered 2QS-1 (82% [95 CI: 75%–88%], 89% [95% CI: 71%–98%], 80% [95% CI: 71%–86%], respectively) and for a positive response to either question (0.80% [95% CI: 72%–86%], 93% [95% CI: 75%–99%], 76% [95% CI: 67%–83%], respectively).

Electronically administered 2QS provided slightly lower accuracy, with comparable sensitivity, but lower specificity (Table S1). Clinic and online scores were calculated separately for diagnostic accuracy given the significant difference in 2QS scores between groups with only small changes observed (Tables S3 and S4).

##### Reliability

Internal consistency measured using Cronbach’s alpha for the two items of the 2QS was α = 0.82 for the total sample, with α = 0.89 for the online sample and α = 0.78 for in-clinic participants completing the survey on a tablet. When clinician administered, Cronbach’s alpha for the two items of the 2QS was α = 0.79 for the total sample, and this was consistent for telehealth and in-clinic subsamples.

##### Validity

Concurrent validity was assessed by correlating a positive response of the clinician-administered 2QS with SCID-5-MDD, PHQ-9 and DASS-21 depression scale (DASS-D; Table S2). The PHQ-9 and DASS-D each demonstrated significant predictive ability for SCID-MDD (Table S5). Pearson’s correlation with SCID-5-MDD was *r* = 0.37, increasing to *r* = 0.54 with subthreshold symptoms added. The correlation for clinician-administered 2QS was *r* = 0.65 with PHQ-9 and *r* = 0.70 with DASS-D. For the telehealth sample, clinician-administered 2QS with SCID-5-MDD was *r* = 0.31, and with subthreshold depression symptoms added, *r* = 0.36. The correlation with the PHQ-9 was *r* = 0.42, and that with DASS-D was *r* = 0.52. For the in-clinic sample, clinician-administered 2QS with SCID-5-MDD was *r* = 0.39, and with subthreshold depression symptoms added, *r* = 0.60. The in-clinic 2QS correlation with for the PHQ-9 was *r* = 0.73, and that with DASS-D was *r* = 0.74. See scale and subscale correlations in Table S2.

## Discussion

This study assessed the clinical utility, feasibility and acceptability of a 2QS for depression, administered in routine neurology consultations. The clinician-administered 2QS performed well, showing excellent ability to accurately detect depression, but with lower ability to accurately exclude it. Clinician administration of the 2QS was feasible, with three-quarters of participants being screened.

The sensitivity of the clinician-administered 2QS to detect MDD in the current study (100%) was equivalent to that reported by Mohr et al.,^22^ who reported sensitivity of 99%. However, we found lower specificity (68%) than the 87% reported by Mohr et al. While Mohr et al. used the SCID interview, it is unclear whether the 2QS was administered within the same telephone administration session, and if so, in what order. This may mean 2QS results were not blinded to the SCID administrator. The current study also differed in that the 2QS was administered in routine clinical consultations, whether in clinic or via telehealth.

We found lower sensitivity and specificity with electronic 2QS administration (sensitivity 93%, specificity 55%), suggesting that delivery mode affects response accuracy. A previous study that similarly administered a survey-based 2QS found comparable sensitivity and specificity (96% and 57%, respectively), albeit not in people with MS.^21^ In our study, participants were significantly more likely to endorse either or both items on the 2QS when administered electronically versus during clinician consultations, which possibly reflects an interviewer effect driven by participants’ perceptions of the interviewer.^31^ In addition, the clinician’s ability to ask clarifying questions, which was not available for tablet/online survey administration, may have been a contributing factor. Indeed, the ability to ask clarifying questions, as opposed to standardised scripting, has previously shown improved response accuracy.^32^

Routine clinician administration of the 2QS was deemed feasible, with an adherence rate of 76%, indicating that it provides a practical depression screening method. Given that only consenting participants were screened, it is likely that standard routine screening would improve screening rates by eliminating manually triggered clinical processes. Despite implementation challenges, the importance of routine depression screening in MS clinics remains. Routine screening has been recommended in MS-management guidelines, and the 2QS should be integrated into these guidelines due to its efficiency and accuracy in clinical practice.^18,20^ Following a positive screen, clinicians should refer their patients to a mental health specialist for diagnosis and treatment, address barriers to mental health treatment and follow up with the referred to specialist to ensure treatment adherence.

### Strengths and limitations

While other studies have assessed depression-screening tools in MS,^33,34^ this is, to our knowledge, the first study to psychometrically evaluate clinician administration of the 2QS in routine consultations. However, there are limitations to consider. Our study was conducted predominantly on individuals diagnosed with relapsing-remitting MS and with mild disability, which may limit generalisability. Participation was voluntary, which may have introduced selection bias. In addition, clinicians were not required to follow up on screening outcomes; instead, participants’ nominated medical practitioners (usually their GP) were notified of symptoms requiring follow-up. Qualitative research by our team indicates that for successful routine depression screening, MS clinical personnel require improved patient information and referral resources to manage positive screens. Thus, further research should assess the feasibility when resources are provided to support clinician follow-up of mental health symptoms.^20^ Finally, screening was only performed at one time-point; test–retest data should be incorporated into future studies to determine the reliability of the 2QS over time.

## Conclusions

This study supports the utility and feasibility of routine clinician administration of the 2QS to accurately screen for MDD. Given that depression is a symptom of MS with substantial socioeconomic and quality-of-life consequences, further efforts to prioritise routine screening are required.

## Supplemental Material

sj-docx-1-msj-10.1177_13524585261435415 – Supplemental material for Utility, validity, feasibility and acceptability of a clinician-administered depression, two-question screening tool for routine multiple sclerosis clinic administration Supplemental material, sj-docx-1-msj-10.1177_13524585261435415 for Utility, validity, feasibility and acceptability of a clinician-administered depression, two-question screening tool for routine multiple sclerosis clinic administration by Lisa Grech, Michelle Allan, David Skvarc, Ruby Hamer, Emily Friedel, Victor Chong, Andrew Giles, Jayashri Kulkarni, Jennifer Neil, Deepa Rajendran, Nevin John, Martin Short, Sally Shaw, Nigel Caswell, Vanessa Fanning and Ernest Butler in Multiple Sclerosis Journal

## Data Availability

Data will be shared by the corresponding author upon reasonable request.
