# Supplementary material for: Utility, validity, feasibility and acceptability of a clinician-administered depression, two-question screening tool for routine multiple sclerosis clinic administration
Source: Mult Scler. 2026 Apr 26;32(11):1205–13. doi: 10.1177/13524585261435415 (PMC13646438; doi:10.1177/13524585261435415)
Supplement: Supplementary file 1 [file sj-docx-1-msj-10.1177_13524585261435415.docx]

**Supplementary Information**

**Table S1.** Accuracy, specificity, and sensitivity of the two-question screening tool for depression

|  | 26% Population Prevalence** | | | | |  | Sample Prevalence Used | | | |  |  |  |
| --- | --- | --- | --- | --- | --- | --- | --- | --- | --- | --- | --- | --- | --- |
| **Depression Measure** | **Sensitivity** | **Specificity** | **Accuracy** | **PPV** | **NPV** |  | **Accuracy** | **PPV** | **NPV** | **LR+** | **LR-** | **Youden/ Cutoff** | **ROC** |
| **2QS** |  |  |  |  |  |  |  |  |  |  |  |  |  |
| ***SCID MDD*** |  |  |  |  |  |  |  |  |  |  |  |  |  |
| Clinician 2QS-1 | 1.00 | 0.73 | 0.80 | 0.56 | 1.00 |  | 0.75 | 0.22 | 1.00 | 3.66 | 0.00 | 0.73 | 0.86 |
| Clinician 2QS-2 | 0.73 | 0.76 | 0.75 | 0.51 | 0.89 |  | 0.75 | 0.19 | 0.97 | 2.97 | 0.36 | 0.49 | 0.74 |
| Clinician 2QS-Any | 1.00 | 0.68 | 0.77 | 0.53 | 1.00 |  | 0.71 | 0.20 | 1.00 | 3.16 | 0.00 | 0.68 | 0.82 |
| Clinician 2QS-Both | 0.73 | 0.81 | 0.79 | 0.57 | 0.89 |  | 0.80 | 0.23 | 0.97 | 3.78 | 0.34 |  | 0.77 |
| Tablet/Online 2QS-1 | 0.93 | 0.60 | 0.69 | 0.45 | 0.96 |  | 0.63 | 0.18 | 0.99 | 2.35 | 0.11 | 0.53 | 0.77 |
| Tablet/Online 2QS-2 | 0.87 | 0.63 | 0.69 | 0.45 | 0.93 |  | 0.65 | 0.17 | 0.98 | 3.32 | 0.21 | 0.50 | 0.75 |
| Tablet/Online 2QS-Any | 0.93 | 0.55 | 0.65 | 0.42 | 0.96 |  | 0.58 | 0.16 | 0.99 | 2.07 | 0.12 | 0.48 | 0.74 |
| Tablet/Online 2QS-Both | 0.87 | 0.68 | 0.73 | 0.49 | 0.94 |  | 0.70 | 0.20 | 0.98 | 2.71 | 0.20 |  | 0.77 |
| ***SCID MDD + Subthreshold*** | |  |  | | |  |  |  |  |  |  |  |  |
| Clinician 2QS-1 | 0.89 | 0.80 | 0.82 | 0.61 | 0.95 |  | 0.81 | 0.49 | 0.97 | 4.37 | 0.14 | 0.69 | 0.84 |
| Clinician 2QS-2 | 0.78 | 0.83 | 0.82 | 0.62 | 0.91 |  | 0.82 | 0.50 | 0.94 | 4.56 | 0.27 | 0.61 | 0.80 |
| Clinician 2QS-Any | 0.93 | 0.76 | 0.80 | 0.57 | 0.97 |  | 0.79 | 0.45 | 0.98 | 3.80 | 0.10 | 0.68 | 0.84 |
| Clinician 2QS-Both | 0.74 | 0.88 | 0.84 | 0.68 | 0.91 |  | 0.85 | 0.57 | 0.94 | 6.07 | 0.30 | 0.62 | 0.81 |
| Tablet/Online 2QS-1 | 0.97 | 0.67 | 0.74 | 0.51 | 0.98 |  | 0.72 | 0.38 | 0.99 | 2.90 | 0.05 | 0.64 | 0.82 |
| Tablet/Online 2QS-2 | 0.90 | 0.69 | 0.74 | 0.50 | 0.95 |  | 0.72 | 0.37 | 0.97 | 2.88 | 0.14 | 0.59 | 0.80 |
| Tablet/Online 2QS-Any | 0.97 | 0.61 | 0.70 | 0.46 | 0.98 |  | 0.67 | 0.34 | 0.99 | 2.46 | 0.05 | 0.58 | 0.79 |
| Tablet/Online 2QS-Both | 0.90 | 0.75 | 0.79 | 0.56 | .096 |  | 0.77 | 0.42 | 0.97 | 3.57 | 0.13 | 0.65 | 0.83 |

**Table S2.** Pearsons correlations between depression scales and subscales for the entire sample.

| Correlations | 1 | 2 | 3 | 4 | 5 | 6 | 7 | 8 | 9 | 10 | 11 | 12 | 13 |
| --- | --- | --- | --- | --- | --- | --- | --- | --- | --- | --- | --- | --- | --- |
| 1. 2QS-1 | -- |  |  |  |  |  |  |  |  |  |  |  |  |
| 2. 2QS-2 | .699^**^ | -- |  |  |  |  |  |  |  |  |  |  |  |
| 3. 2QS-Any | -.884^**^ | -.839^**^ | -- |  |  |  |  |  |  |  |  |  |  |
| 4. 2QS-Both | -.834^**^ | -.879^**^ | .738^**^ | -- |  |  |  |  |  |  |  |  |  |
| 5. Clinician2QS1 | -.677^**^ | -.548^**^ | .608^**^ | .634^**^ | -- |  |  |  |  |  |  |  |  |
| 6. Clinician2QS2 | -.616^**^ | -.689^**^ | .591^**^ | .733^**^ | .653^**^ | -- |  |  |  |  |  |  |  |
| 7. Clinician2QSAny | -.703^**^ | -.611^**^ | .652^**^ | .679^**^ | .881^**^ | .808^**^ | -- |  |  |  |  |  |  |
| 8. Clinician2QSBoth | -.620^**^ | -.658^**^ | .576^**^ | .721^**^ | .780^**^ | .878^**^ | .709^**^ | -- |  |  |  |  |  |
| 9. SCID Depression | -.297^**^ | -.276^**^ | .266^**^ | .314^**^ | .404^**^ | .280^**^ | .370^**^ | .329^**^ | -- |  |  |  |  |
| 10. SCID Depression Subclin | -.350^**^ | -.331^**^ | .317^**^ | .371^**^ | .358^**^ | .410^**^ | .365^**^ | .422^**^ | -0.09 | -- |  |  |  |
| 11. SCIDDepression + Subclin | -.481^**^ | -.451^**^ | .433^**^ | .509^**^ | .562^**^ | .519^**^ | .544^**^ | .562^**^ | .651^**^ | .697^**^ | -- |  |  |
| 12. PHQ9 | -.645^**^ | -.642^**^ | .605^**^ | .699^**^ | .640^**^ | .636^**^ | .639^**^ | .657^**^ | .470^**^ | .375^**^ | .624^**^ | -- |  |
| 13. DASS-Depression | -.699^**^ | -.662^**^ | .691^**^ | .686^**^ | .697^**^ | .619^**^ | .675^**^ | .676^**^ | .441^**^ | .337^**^ | .574^**^ | .825^**^ | -- |
| **Correlation is significant at the 0.01 level (2-tailed). | | | | | | | | | | | | | |

**Table S3.** Participants attending appointments in-clinic

|  | 26% Population Prevalence** | | | | |  | Sample Prevalence Used | | | |  |  |  |
| --- | --- | --- | --- | --- | --- | --- | --- | --- | --- | --- | --- | --- | --- |
| **Depression Measure** | **Sensitivity** | **Specificity** | **Accuracy** | **PPV** | **NPV** |  | **Accuracy** | **PPV** | **NPV** | **LR+** | **LR-** | **Youden/ Cutoff** | **ROC** |
| **2QS** |  |  |  |  |  |  |  |  |  |  |  |  |  |
| ***SCID MDD*** |  |  |  |  |  |  |  |  |  |  |  |  |  |
| Clinician 2QS-1 | 1.00 | 0.71 | 0.79 | 0.54 | 1.00 |  | 0.74 | 0.25 | 1.00 | 3.44 | 0.00 | 0.71 | 0.86 |
| Clinician 2QS-2 | 0.82 | 0.69 | 0.72 | 0.48 | 0.92 |  | 0.70 | 0.23 | 0.97 | 2.64 | 0.26 | 0.52 | 0.76 |
| Clinician 2QS-Any | 1.00 | 0.67 | 0.75 | 0.51 | 1.00 |  | 0.70 | 0.23 | 1.00 | 3.00 | 0.00 | 0.67 | 0.83 |
| Clinician 2QS-Both | 0.78 | 0.80 | 0.79 | 0.57 | 0.91 |  | 0.79 | 0.27 | 0.97 | 3.81 | 0.28 | 0.57 | 0.79 |
| Tablet/Online 2QS-1 | 1.00 | 0.55 | 0.66 | 0.44 | 1.00 |  | 0.59 | 0.20 | 1.00 | 2.20 | 0.00 | 0.55 | 0.77 |
| Tablet/Online 2QS-2 | 0.91 | 0.54 | 0.64 | 0.41 | 0.95 |  | 0.58 | 0.18 | 0.98 | 2.02 | 0.15 | 0.46 | 0.73 |
| Tablet/Online 2QS-Any | 1.00 | 0.46 | 0.60 | 0.40 | 1.00 |  | 0.52 | 0.17 | 1.00 | 1.86 | 0.00 | 0.46 | 0.73 |
| Tablet/Online 2QS-Both | 0.92 | 0.63 | 0.70 | 0.47 | 0.96 |  | 0.66 | 0.22 | 0.99 | 2.48 | 0.13 | 0.55 | 0.77 |
| ***SCID MDD + Subthreshold*** | |  |  | | |  |  |  |  |  |  |  |  |
| Clinician 2QS-1 | 0.91 | 0.80 | 0.83 | 0.61 | 0.96 |  | 0.82 | 0.56 | 0.97 | 4.55 | 0.11 | 0.71 | 0.86 |
| Clinician 2QS-2 | 0.82 | 0.84 | 0.83 | 0.64 | 0.93 |  | 0.83 | 0.58 | 0.93 | 5.03 | 0.22 | 0.66 | 0.83 |
| Clinician 2QS-Any | 0.95 | 0.76 | 0.81 | 0.59 | 0.98 |  | 0.80 | 0.52 | 0.98 | 4.02 | 0.06 | 0.72 | 0.86 |
| Clinician 2QS-Both | 0.77 | 0.89 | 0.86 | 0.71 | 0.92 |  | 0.86 | 0.65 | 0.93 | 6.87 | 0.26 | 0.66 | 0.83 |
| Tablet/Online 2QS-1 | 1.00 | 0.63 | 0.72 | 0.48 | 1.00 |  | 0.71 | 0.43 | 1.00 | 2.69 | 0.00 | 0.63 | 0.82 |
| Tablet/Online 2QS-2 | 0.92 | 0.62 | 0.70 | 0.46 | 0.96 |  | 0.68 | 0.40 | 0.97 | 2.41 | 0.12 | 0.54 | 0.77 |
| Tablet/Online 2QS-Any | 1.00 | 0.53 | 0.65 | 0.43 | 1.00 |  | 0.63 | 0.37 | 1.00 | 2.14 | 0.00 | 0.53 | 0.77 |
| Tablet/Online 2QS-Both | 0.92 | 0.71 | 0.77 | 0.53 | 0.96 |  | 0.76 | 0.47 | 0.97 | 3.21 | 0.11 | 0.64 | 0.82 |

**Table S4.**  Participants attending appointments via telehealth

|  | 26% Population Prevalence** | | | | |  | Sample Prevalence Used | | | | |  |  |
| --- | --- | --- | --- | --- | --- | --- | --- | --- | --- | --- | --- | --- | --- |
| **Depression Measure** | **Sensitivity** | **Specificity** | **Accuracy** | **PPV** | **NPV** |  | **Accuracy** | **PPV** | **NPV** | **LR+** | **LR-** | **Youden/ Cutoff** | **ROC** |
| **2QS** |  |  |  |  |  |  |  |  |  |  |  |  |  |
| ***SCID MDD*** |  |  |  |  |  |  |  |  |  |  |  |  |  |
| Clinician 2QS-1 | 1.00 | 0.76 | 0.82 | 0.60 | 1.00 |  | 0.77 | 0.15 | 1.00 | 4.18 | 0.00 | 0.76 | 0.88 |
| Clinician 2QS-2 | 0.50 | 0.78 | 0.71 | 0.45 | 0.82 |  | 0.77 | 0.09 | 0.97 | 2.30 | 0.64 | 0.28 | 0.64 |
| Clinician 2QS-Any | 1.00 | 0.72 | 0.79 | 0.55 | 1.00 |  | 0.73 | 0.13 | 1.00 | 3.54 | 0.00 | 0.72 | 0.86 |
| Clinician 2QS-Both | 0.50 | 0.83 | 0.74 | 0.50 | 0.82 |  | 0.81 | 0.11 | 0.97 | 2.88 | 0.61 | 0.33 | 0.66 |
| Tablet/Online 2QS-1 | 0.67 | 0.71 | 0.70 | 0.44 | 0.86 |  | 0.70 | 0.11 | 0.98 | 2.27 | 0.47 | 0.37 | 0.69 |
| Tablet/Online 2QS-2 | 0.67 | 0.78 | 0.75 | 0.51 | 0.87 |  | 0.77 | 0.13 | 0.98 | 2.97 | 0.43 | 0.44 | 0.72 |
| Tablet/Online 2QS-Any | 0.67 | 0.71 | 0.70 | 0.44 | 0.86 |  | 0.70 | 0.11 | 0.98 | 2.27 | 0.47 | 0.37 | 0.69 |
| Tablet/Online 2QS-Both | 0.67 | 0.78 | 0.75 | 0.51 | 0.87 |  | 0.77 | 0.13 | 0.98 | 2.97 | 0.43 | 0.44 | 0.72 |
| ***SCID MDD + Subthreshold*** | |  |  | | |  |  |  |  |  |  |  |  |
| Clinician 2QS-1 | 0.80 | 0.79 | 0.79 | 0.57 | 0.92 |  | 0.79 | 0.31 | 0.97 | 3.82 | 0.25 | 0.59 | 0.80 |
| Clinician 2QS-2 | 0.60 | 0.81 | 0.76 | 0.53 | 0.85 |  | 0.79 | 0.27 | 0.95 | 3.23 | 0.49 | 0.41 | 0.71 |
| Clinician 2QS-Any | 0.80 | 0.74 | 0.52 | 0.91 | 0.76 |  | 0.75 | 0.27 | 0.97 | 3.13 | 0.27 | 0.54 | 0.77 |
| Clinician 2QS-Both | 0.60 | 0.86 | 0.79 | 0.60 | 0.86 |  | 0.83 | 0.33 | 0.95 | 4.30 | 0.46 | 0.46 | 0.73 |
| Tablet/Online 2QS-1 | 0.80 | 0.73 | 0.75 | 0.51 | 0.91 |  | 0.74 | 0.21 | 0.98 | 2.99 | 0.27 | 0.53 | 0.77 |
| Tablet/Online 2QS-2 | 0.80 | 0.80 | 0.80 | 0.59 | 0.92 |  | 0.80 | 0.27 | 0.98 | 4.07 | 0.25 | 0.60 | 0.80 |
| Tablet/Online 2QS-Any | 0.80 | 0.73 | 0.75 | 0.51 | 0.91 |  | 0.74 | 0.21 | 0.98 | 2.99 | 0.27 | 0.53 | 0.77 |
| Tablet/Online 2QS-Both | 0.80 | 0.80 | 0.80 | 0.59 | 0.92 |  | 0.80 | 0.27 | 0.98 | 4.07 | 0.25 | 0.60 | 0.80 |

**Table S5.** Accuracy, specificity, and sensitivity of the PHQ-9 and DASS-21 for depression

|  | 26% Population Prevalence** | | | | |  | Sample Prevalence Used | | | | |  | |
| --- | --- | --- | --- | --- | --- | --- | --- | --- | --- | --- | --- | --- | --- |
| **Depression Measure** | **Sensitivity** | **Specificity** | **Accuracy** | **PPV** | **NPV** |  | **Accuracy** | **PPV** | **NPV** | **LR+** | **LR-** | **Youden/ Cutoff** | **ROC** |
| **PHQ-9** |  |  |  |  |  |  |  |  |  |  |  |  |  |
| **SCID MDD** |  |  |  |  |  |  |  |  |  |  |  |  | 0.91 |
| PHQ9 ≧8 | 1.00 | 0.66 | 0.75 | 0.51 | 1.00 |  | 0.69 | 0.2 | 0.1 | 2.96 | 0.00 | 0.65 |  |
| PHQ9 ≧10 | 0.79 | 0.79 | 0.79 | 0.57 | 0.91 |  | 0.79 | 0.24 | 0.98 | 3.73 | 0.27 | 0.58; Mod-Ex Severe* |  |
| **DASS-D** |  |  |  |  |  |  |  |  |  |  |  |  |  |
| **SCID MDD** |  |  |  |  |  |  |  |  |  |  |  |  | 0.89 |
| DASS-D ≧6 | 0.93 | 0.75 | 0.79 | 0.56 | 0.97 |  | 0.93 | 0.25 | 0.68 | 3.67 | 0.10 | 0.68 |  |
| DASS-D ≧7 | 0.86 | 0.80 | 0.81 | 0.60 | 0.94 |  | 0.80 | 0.27 | 0.99 | 4.18 | 0.18 | 0.65; Mod-Ex Severe* |  |

**Figure S1**. STARD diagram to report the flow of participants through the study
